# Intrathecal morphine is associated with reduction in postoperative opioid requirements and improvement in postoperative analgesia in patients undergoing open liver resection

**DOI:** 10.1186/s12871-020-01113-8

**Published:** 2020-08-19

**Authors:** Jefferson Tang, Leonid Churilov, Chong Oon Tan, Raymond Hu, Brett Pearce, Luka Cosic, Christopher Christophi, Laurence Weinberg

**Affiliations:** 1 Department of Anaesthesia, Austin Health, Heidelberg, Victoria Australia; 2 Melbourne Medical School, University of Melbourne, Heidelberg, Victoria Australia; 3 Department of Surgery, University of Melbourne, Austin Health, Heidelberg, Victoria Australia

**Keywords:** Analgesia, Liver resection, Hospital costs, Intrathecal morphine, Enhanced recovery after surgery

## Abstract

**Background:**

Our study aimed to test the hypothesis that the addition of intrathecal morphine (ITM) results in reduced postoperative opioid use and enhanced postoperative analgesia in patients undergoing open liver resection using a standardized enhanced recovery after surgery (ERAS) protocol with multimodal analgesia.

**Methods:**

A retrospective analysis of 216 adult patients undergoing open liver resection between June 2010 and July 2017 at a university teaching hospital was conducted. The primary outcome was the cumulative oral morphine equivalent daily dose (oMEDD) on postoperative day (POD) 1. Secondary outcomes included postoperative pain scores, opioid related complications, and length of hospital stay. We also performed a cost analysis evaluating the economic benefits of ITM.

**Results:**

One hundred twenty-five patients received ITM (ITM group) and 91 patients received usual care (UC group). Patient characteristics were similar between the groups. The primary outcome - cumulative oMEDD on POD1 - was significantly reduced in the ITM group. Postoperative pain scores up to 24 h post-surgery were significantly reduced in the ITM group. There was no statistically significant difference in complications or hospital stay between the two study groups. Total hospital costs were significantly higher in the ITM group.

**Conclusion:**

In patients undergoing open liver resection, ITM in addition to conventional multimodal analgesic strategies reduced postoperative opioid requirements and improved analgesia for 24 h after surgery, without any statistically significant differences in opioid-related complications, and length of hospital stay. Hospital costs were significantly higher in patients receiving ITM, reflective of a longer mandatory stay in intensive care.

**Trial registration:**

Registered with the Australian New Zealand Clinical Trials Registry (ANZCTR) under ACTRN12620000001998.

## Background

Perioperative analgesia is critical for maximising patient satisfaction and recovery outcomes in surgery. The optimal postoperative analgesic technique for patients undergoing open liver resection is controversial. Continuous thoracic epidural analgesia has been considered the cornerstone analgesic modality, however limitations of its use in this setting include risk of epidural haematoma (in the context of coagulopathy associated with postoperative hepatic insufficiency), prolonged motor block limiting mobilisation, urinary retention, and hypotension [1–3]. Epidural analgesia is furthermore a labor intensive and more technically complicated intervention. Indeed, a large international and multicenter landmark randomized controlled trial (RCT) found that most adverse morbid outcomes in high-risk patients undergoing major abdominal surgery are not reduced by the use of epidural analgesia [4]. The Enhanced Recovery After Surgery (ERAS) Society guidelines now strongly advocate that routine thoracic epidural analgesia cannot be recommended in open liver surgery for ERAS patients and that intrathecal opiates can be good alternatives when combined with multimodal analgesia [5].

Single shot intrathecal morphine (ITM) has recently emerged as a promising alternative practice yielding better patient outcomes [6–8]. Intrathecal anaesthesia is a simpler and quicker alternative neuraxial technique with a lower rate of technical failure [7]. Similar to epidural analgesia, intrathecal morphine has been demonstrated to improve postoperative pain scores and reduce postoperative rescue analgesia requirements compared to intravenous opioid analgesia [8]. As such, a growing number of hospitals worldwide have adopted ITM as a preferred choice for perioperative analgesia for major open hepato-pancreatic-biliary surgery [6]. However, the benefits of ITM compared to conventional multimodal analgesic strategies for major open liver surgery remain unclear. Therefore, we conducted a retrospective observational study to determine if patients undergoing open liver resection who receive ITM in additional to receiving a standardized ERAS protocol have better postoperative analgesia compared to patients receiving ERAS alone. We hypothesize that for patients undergoing open liver resection using a standardized enhanced recovery after surgery protocol with multimodal analgesia, the addition of ITM results in less postoperative opioid use and enhanced postoperative analgesia.

## Methods

Following prospective ethics approval by the Austin Health Human Research Ethics Committee (HREC no: LNR/18/Austin/79), we conducted a retrospective analysis of adult patients who underwent major open liver resection between July 2010 and June 2017 using a standardized ERAS protocol. All patients underwent surgery at the Austin Hospital, a university hospital in Melbourne, Australia with a dedicated high volume hepato-pancreatic-biliary and liver transplant centre. Eligible patients were identified by International Statistical Classification of Diseases (ICD) codes that included the following surgical categories: i.) excision of lesion of liver, ii.) segmental resection of liver, iii.) lobectomy of liver, iv.) trisegmental resection of liver, and v.) segmental resection of liver for trauma. In order to ensure a homogeneous patient cohort, we excluded patients receiving epidural analgesia, patients undergoing laparoscopic liver resection, and patients undergoing deroofing of liver cyst or liver biopsy. We also excluded patients with a history of chronic opioid use (defined as near-daily use of > 60 mg oral morphine equivalent) for 8 weeks or longer. A team of experienced high-volume surgeons (*n* = 5) and anaesthetists (*n* = 6) provided perioperative care based on a standardized liver enhanced recovery after surgery programme. As part of this protocol, all patients received an opioid based patient-controlled analgesia device for postoperative analgesia.

### Key outcomes

The primary outcome was cumulative oral morphine equivalent daily dose in milligrams (oMEDD) on POD 1, adjusted for the following priori chosen variables: major resection, patient age, Charlson Comorbidity Index (CCI), duration of surgery, intraoperative oMEDD use, adjunct intrathecal clonidine, adjunct intrathecal bupivacaine, intraoperative ketamine and postoperative ketamine. These factors were chosen due to their potential influence on total morphine requirements after surgery. Secondary outcomes included average and maximum pain at rest and on movement over the first 24 postoperative hours, oMEDD use and postoperative pain scores in the PACU and on postoperative days 0, 2 and 3. We measured opioid related side effects, length of hospital stay, and performed a costs analysis (including readmissions within 30 postoperative days) for both groups of patients.

### Definitions

oMEDD amounts were calculated using the Opioid Dose Equivalence document endorsed by the Faculty of Pain Medicine, Australian and New Zealand College of Anaesthetists [9]. Length of hospital stay was determined by the period from completion of surgery to discharge. Time to full ward diet was defined as the period from completion of surgery to the first mention of tolerating full ward diet in the patient medical records. Time to first oral opioid use was defined as the period from completion of surgery to the first administration of oral opioid after surgery. Daily postoperative pain scores were measured and recorded by a dedicated Acute Pain Service using the Numerical Rating Scale (NRS). The NRS is a single 11-point numeric scale in which a respondent selects a whole number (integers from 0 to 10) that best reflects the intensity of their pain. A score of 0 indicates no pain, whereas a score of 10 indicates extreme or the worst pain imaginable [10]. Duration of surgery was defined from skin incision until the final stitch for wound closure. Major resection was defined as 4 segments or greater; minor resection was defined as 3 segments or less.

Complications were defined as any deviation from the normal postoperative course, guided by the European Perioperative Clinical Outcome definitions [11]. Complications were recorded by two independent clinicians, and then graded using Clavien-Dindo Classification - a widely used and validated approach to surgical outcome assessment that assigns severity grades to surgical complications [12]. In case of disagreement on grading by the two assessors, the case was discussed with a third author.

Costs related to the index hospital admission and any consequent readmission within 30 postoperative days were included. Costs related to the preoperative and perioperative course were not considered. Allocation of costs was done based on service volume, and costs were calculated using an activity-based costing methodology. Raw costing data was obtained from the hospital’s business intelligent unit, and then allocated into categories based on individual itemisation codes for costs incurred during admission. These categories included ‘intensive care unit’, ‘medical’ (for example medical consults, allied health, pathology, blood products, and radiology), ‘pharmacy’, and ‘ward’ costs. For detailed cost analysis of complication incidence and severity, cost centres were further separated into ‘allied health’ (for example physiotherapy, speech pathology, dietician), ‘blood products’ (for example albumin, packed red cells), ‘intensive care unit’, ‘pathology’ (for example tissue diagnosis, blood testing), ‘pharmacy’ (drug dispensing), ‘radiology’ (for example scans, radiological procedures), and ‘ward’ (for example hospital bed, nursing, catering). Only in-hospital costs were considered, with both direct and indirect costs assessed to produce a total cost for each patient. Costs are displayed as medians and interquartile ranges to more accurately reflect the economic burden placed upon healthcare providers by catering for outliers. Costs were inflated to 2018 dollars based upon the average Australian Consumer Price Index from 2010 to 2017 inclusive, as reported by the Reserve Bank of Australia [13]. The average Consumer Price Index was applied pro rata to each patient based on the number of days between the admission date and the 1st of January 2018, to ensure all costs were inflated as accurately as possible reducing error in comparison. Conversion to the United States Dollar was completed using the market rate on the 1st of January 2018.

### Data collection

Data was extracted from the patient’s electronic medical records and the Hospital’s computerized laboratory results. Austin Health utilizes Cerner® electronic health records that allows comprehensive electronic data capture and access to patient health information in the perioperative setting.

Preoperative data collected included gender, age, body mass index, American Society of Anesthesiologists (ASA) class, principle diagnosis, surgical procedure, segments resected, and Charlson Comorbidity Index (CCI). The ASA score provides a simple categorisation of a patient’s physiological status before surgery and is useful in predicting perioperative morbidity and mortality [14]. The CCI is a validated method for classifying comorbid conditions and subsequently estimating the risk of mortality from comorbid diseases [15]. We additionally collected information on whether the patient had smoked in the 1 year prior to the operation, preoperative biochemistry, liver function test and full blood examination results.

Intraoperative data collected included duration of surgery, type of resection, and whether the resection was “major” or “minor”. We collected the dose of intrathecal morphine administered just prior to the commencement of the surgery, type and amount of intraoperative analgesia delivered, and types and amounts of fluids and blood products used intraoperatively.

Postoperative data collected included length of stay, duration of PCA use, time to full ward diet, time to first oral opioid use, and time in the intensive care unit (ICU). We also collected data on postoperative analgesia agents and anti-emetics administered in the post-anaesthesia care unit (PACU), on postoperative day 0 (day of surgery) and postoperative days 1 to 3. The number and severity of postoperative complications, worst sedation scores and pain scores at rest and on movement in the PACU and POD 0–3 were also collected from the patient’s electronic medical records.

### Statistical methodology

For the primary and the key secondary outcomes, we used quantile regression modelling adjusted for the following a-priori defined covariates: major resection, patient age, Charlson Comorbidity Index, duration of surgery, intraoperative oMEDD use, adjunct intrathecal clonidine, adjunct intrathecal bupivacaine, intraoperative ketamine and postoperative ketamine. Quantile regression models were used due to the violation of the normality of residuals assumption required for standard linear regression and lack of suitable transformations to satisfy these assumptions. Quantile regression models the association between a set of input variables and specific percentiles (or quantiles) of the outcome variable and estimates differences in the quantiles of the outcome variable between standard care and ITM groups. For example, a median (50th percentile) regression of cumulative oMEDD use at 24 h on ITM use estimates the difference in the median oMEDD use at 24 h between standard care and ITM groups adjusted for the selected covariates. For each outcome, we included three quantile regression models: the 25th percentile, the 50th percentile (median), and the 75th percentile. Standard assessment of collinearity was conducted using variance inflation factors (VIF) and condition number. For all other outcomes, continuous data was summarized as medians and interquartile range (IQR) and compared using the Mann-Whitney U test. Categorical variables were summarized as counts (proportions) and compared using the chi-squared test or Fisher’s Exact test, as appropriate. Statistical analysis was performed using statistical software RStudio (Boston, MA, USA). Figures were constructed using Prism 6.0 GraphPad software (La Jolla, CA, USA).

## Results

Three hundred thirty-five patients underwent liver resection at the Austin Hospital between July 2010 and June 2017. Two hundred sixteen patients satisfied the inclusion criteria. Of these, 91 patients received usual care (Usual care group) and 125 patients received ITM in addition to usual care (ITM group). Data collection was complete for 216 (100%) patients. Reason for patients not satisfying inclusion criteria included: epidural analgesia (*n* = 4), laparoscopic liver resection (*n* = 106), deroofing of a liver cyst (*n* = 5), and liver biopsy whilst staging pancreatic cancer without sufficient parenchymal resection (*n* = 4).

The median (IQR) patient age was 60 (51:67) years. There were 125 (58%) males and 91 (42%) females. Median (IQR) body mass index (BMI), ASA score and CCI were 26.15 (23.1:30.4) kg/m^2^, 3 (2:3), and 7 (4:8) respectively. The principal diagnosis and indication for resection was benign in 33 (15%) patients and malignant in 183 (85%) patients. 32 (15%) patients received chemotherapy within 3 months before surgery. 153 (71%) patients underwent a ‘minor’ (defined as 3 segments or less) liver resection, and the remaining 63 (29%) patients underwent a ‘major’ (defined as 4 segments or more) liver resection. The range of procedures performed included 35 (16%) right hepatectomies, 17 (8%) left hepatectomies, 2 (1%) central hepatectomies, 137 (63%) segmental resections (3 segments or less), and 25 (12%) extended left or right hepatectomies (5 segments or more). Baseline patient characteristics between the ITM and usual care groups is summarized in Table 1.

**Table 1:** Baseline characteristics of patients receiving intrathecal morphine (ITM) or Usual care. Data presented as median (interquartile range) or number of patients (proportion)

	ITM (***n*** = 125)	Usual care (***n*** = 91)	***P***-value
**Age (years)**	61 (52.5:68.5)	59 (49:66)	0.26
**Gender (male**: **female)**	81 (65%): 44 (35%)	45 (49%): 46 (51%)	0.03
**Body mass index (kg/m**^2^**)**	25.9 (22.8:30.6)	26.2 (23.4:30.1)	0.71
**Charlson comorbidity index**	6.5 (4.0:8.0)	7.0 (4.0:8.0)i	0.99
**Preoperative chemotherapy within 3 months**	21 (16.8%)	11 (12.1%)	0.44
**AMERICAN SOCIETY OF ANAESTHESIOLOGISTS SCORE**			
1	1 (0.8%)	2 (2.2%)	0.72
2	44 (35.2%)	35 (38.5%)	
3	77 (61.6%)	51 (56%)	
4	3 (2.4%)	3 (3.3%)	
**BASELINE BIOCHEMISTRY**			
Haemoglobin (g/L)	143 (129.5:150.5)	131 (122:146)	0.005
White cell count (× 10^9^/L)	7 (5:8)	6 (5:8)	0.05
Platelet count (× 10^9^/L)	220 (158:273.3)	222 (173.5:280)	0.52
Urea (mg/dL)	6 (4.5:7)	6 (4:7)	0.53
Creatinine (μmol/L)	76.5 (63.25:87)	71.5 (61.25:88)	0.34
Estimated glomerular filtration rate (mL/min/1.73m^2^)	86 (75:90)	90 (74.5:90)	0.6
Alanine transaminase (U/L)	49 (33.5:97)	57.5 (41.5:88.25)	0.67
Aspartate transaminase (U/L)	53 (47:85.5)	68 (64:113)	0.24
Alkaline phosphatase (U/L)	147 (94:201.5)	176 (19.3:246.5)	0.33
Gamma-glutamyltransferase (U/L)	109 (65.75:157.5)	125 (72.5:220)	0.72
Albumin (g/L)	39 (35:42)	37.5 (34:39)	0.08
Bilirubin (μmol/L)	11.5 (6.25:16.75)	12 (7.5:21)	0.54
Activated partial thromboplastin time (s)	27 (24:29)	28 (25:30)	0.17
International normalized ratio	1 (1:1)	1 (1:1)	0.4
**PATHOLOGY**			
**Benign**	**13 (10.4%)**	**20 (21.9%)**	0.17
Adenoma	8 (6.4%)	8 (8.8%)	
Haemangioma	1 (0.8%)	2 (2.2%)	
Other liver pathology	4 (3.2%)	10 (11.0%)	
**Malignant**	**112 (89.6%)**	**71 (78.1%)**	
Hepatocellular carcinoma	36 (28.8%)	29 (31.9%)	
Cholangiocarcinoma	14 (11.2%)	5 (5.5%)	
Gallbladder tumor	4 (3.2%)	2 (2.2%)	
Metastatic colorectal	58 (46.4%)	35 (38.5%)	
**COMPLEXITY OF RESECTION**			
Minor resection (≤ 3 sections)	85 (68.0%)	68 (74.7%)	0.29
Major resection (≥ 4 sections)	40 (32.0%)	23 (25.3%)	
**TYPE OF SURGERY**			
Right hepatectomy	23 (18.4%)	12 (13.2%)	0.53
Left hepatectomy	9 (7.2%)	8 (8.8%)	
Central hepatectomy	2 (1.6%)	0 (0%)	
Segmental resection (< 3 segments)	76 (60.8%)	61 (67.0%)	
Extended left or right hepatectomy (> 5+ segments)	15 (12%)	10 (11.0%)	

### Key outcomes

**Fig. 1:**
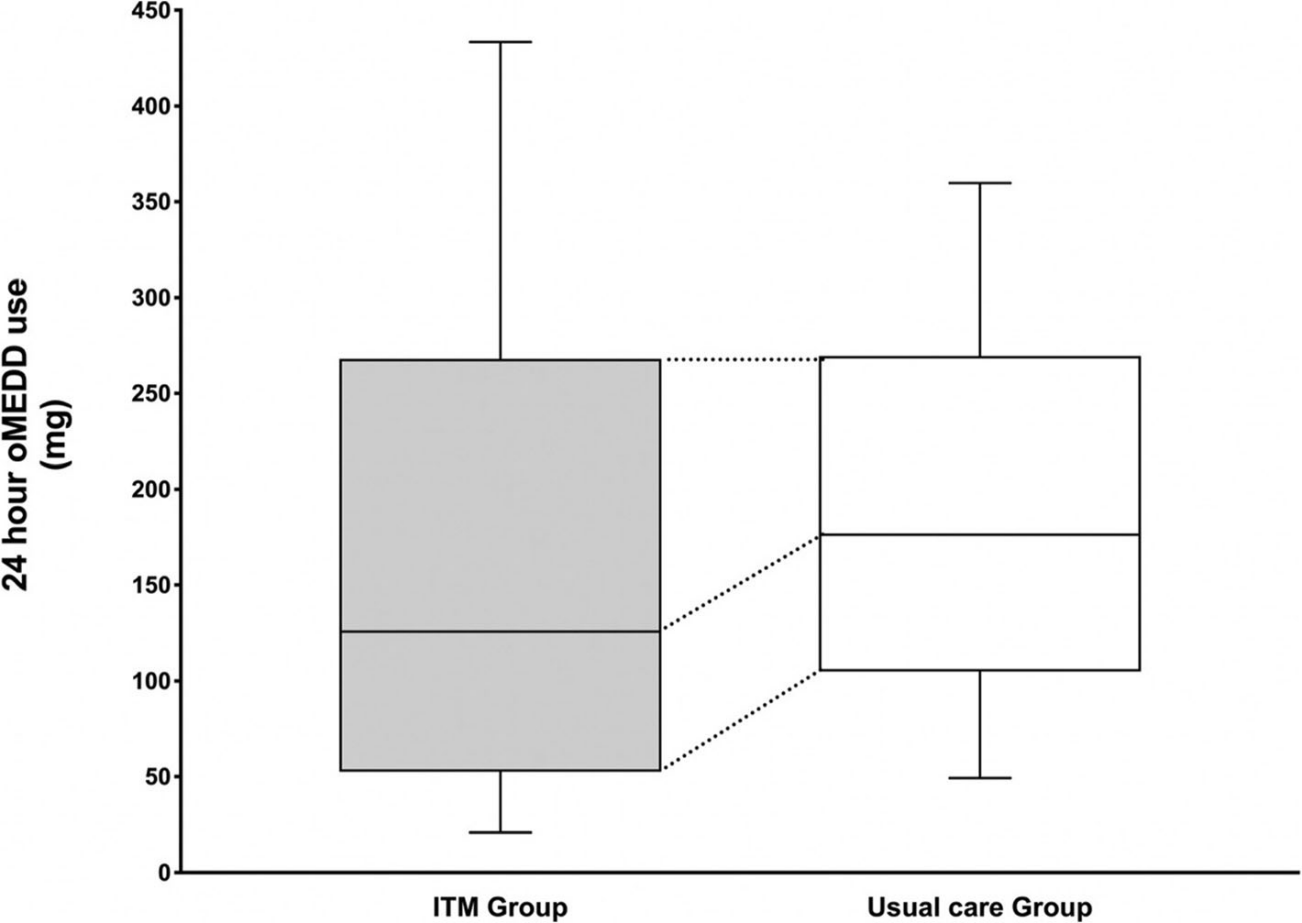
Boxplots of oral morphine equivalent daily dose (oMEDD) use at 24-h post-surgery in patients of patients receiving intrathecal morphine (ITM) or Usual care

The median (IQR) cumulative oMEDD use on postoperative day 1 was 126.7 (53:268) mg in the ITM group vs. 176.3 (105:270) mg in the usual care group (*p* = 0.04). Whilst patients in the ITM group required less morphine compared to the control group, the effect size of this difference depended on their distribution quartile. Patients in the 25th percentile required 55.7 mg less morphine (95% CI: 25.6 to 88.4; *p* = 0.00025); 50th percentile 53.3 mg less morphine (95% CI:-22.1 to 98.0; *p* = 0.058), and 75th percentile 13.9 mg more morphine (95% CI: − 53.1 to 42.4; *p* = 0.65). A boxplot of the cumulative oMEDD consumptions on postoperative day 1 is presented in Fig. 1.

Patients in the ITM group reported lower pain scores at rest and on movement over the first 24 postoperative hours (see Table 2). Pain scores at rest and in the 25th percentile showed the most statistically significant benefit from ITM.

**Table 2:** Differences in morphine consumption and pain scores 24-h after surgery. Data presented as quartile differences (95% confidence interval) for the outcome variables between patients receiving receiving intrathecal morphine and Usual care. Adjustments for major resection, patient age, Charlton Comorbidity Index, duration of surgery, intraoperative oMEDD use, adjunct intrathecal clonidine, adjunct intrathecal bupivacaine, intraoperative ketamine and postoperative ketamine

		25th percentile		50th percentile		75th percentile	
		Adjusted	Unadjusted	Adjusted	Unadjusted	Adjusted	Unadjusted
**Cumulative oMEDD use at 24 h (mg)**	Difference 95% CI *p*-value	55.7 mg 25.6 to 88.4 0.00025	52.3 mg 35.3 to 77.9 0.00015	53.3 mg − 22.1 to 98.0 0.058	50.7 mg 13.4 to 87.8 0.03	−13.9 mg −53.1 to 42.4 0.65	2.9 mg − 62.0 to 47.7 0.94
**Average pain at rest over 24 h**	Difference 95% CI *p*-value	0.99 0.3 to 1.2 0.0005	1 −0.6 to 1.0 0.48	0.38 0.04 to 1.1 0.37	0 0.0 to 3.9 1.0	0.009 −0.8 to 0.5 0.98	0 −2.1 to 1.8 1.0
**Average pain on movement over 24 h**	Difference 95% CI *p*-value	0.9 0.1 to 1.5 0.09	1 1.0 to 2.1 1.0	0.4 − 0.01 to 1.4 0.33	1 − 0.9 to 1.0 0.37	0.2 − 0.3 to 1.1 0.58	1 − 1.6 to 2.1 0.48
**Maximum pain on movement over 24 h**	Difference 95% CI *p*-value	0.9 − 0.08 to 2.3 0.18	2 −1.6 to 2.0 0.15	0.37 − 0.4 to 2.1 0.35	1 −0.9 to 4.9 0.37	0.4 − 0.1 to 1.0 0.26	0 0.0 to 3.6 1.0

### Intraoperative outcomes

For patients receiving ITM, the minimum, median (IQR), and maximum dose of intrathecal morphine delivered were 150 μg, 300 μg (250:400), and 500 μg, respectively. To enhance or lengthen the duration of intrathecal analgesia, intrathecal bupivacaine and clonidine were administered with the ITM in 26 (21%) and 13 (10%) patients respectively. The median (IQR) doses of intrathecal clonidine and bupivacaine were 50 μg (30:15) and 10 mg (2:15), respectively. All patients received additional intraoperative systemic opioids (Table 3). The median (IQR) IV morphine equivalent of intraoperative opioid received was 29.8 mg (20.0:38.0). No statistically significant difference between the ITM group and the usual care group in total operative time or perioperative fluid use and blood product administration were identified. Patients in the ITM group received less intraoperative opioid compared to patients in the usual care group: median (IQR) 26.7 mg (20.0:33.3) vs. 33.3 mg (20.0:46.7) IV morphine equivalent, *p* = 0.001. The ITM group received less intraoperative ketamine: median (IQR) 36 mg (20:62.5) vs. 62.5 mg (38.8:89.5) in the usual care group, *p* = 0.003. A higher number of patients in the ITM group required intraoperative ondansetron (51% vs. 32%, *p* = 0.005).

**Table 3:** Perioperative analgesia use and postoperative morphine consumption of patients receiving intrathecal morphine (ITM) or Usual care. Data presented as median (interquartile range) or number of patients (proportion)

	ITM (***n*** = 125)	Usual care (***n*** = 91)	***P***-value
Total operative time (minutes)	250 (196.5:338)	232 (180:310)	0.08
**INTRAOPERATIVE ANALGESIA**			
Patients receiving opioid	125 (100%)	91 (100%)	> 0.99
IV morphine equivalent (mg)	26.7 (20:33.3)	33.3 (20:46.7)	0.001
Patients receiving paracetamol	44 (35.2%)	27 (29.7%)	0.46
Median amount (mg)	1 (1:1)	1 (1:1)	0.15
Patients receiving ketamine	50 (40%)	26 (28.6%)	0.09
Median amount (mg)	36 (20:62.5)	62.5 (38.75:89.5)	0.003
Patients receiving clonidine	10 (8%)	8 (8.8%)	> 0.99
Median amount (mg)	75 (45:150)	75 (75:97.5)	0.61
Patients receiving IV lignocaine	7 (5.6%)	4 (4.4%)	0.76
Median amount (mg)	220 (150:660)	389 (364.5:467.5)	0.32
Patients receiving ondansetron	64 (51.2%)	29 (31.9%)	0.005
Median amount (mg)	4 (4:4)	4 (4:4)	0.31
Patients receiving metoclopramide	5 (4%)	2 (2.2%)	0.70
Median amount (mg)	20 (15:20)	20 (20:20)	> 0.99
Patients receiving naloxone	2 (1.6%)	3 (3.3%)	0.65
Median amount (μg)	110 (20:200)	100 (80:200)	> 0.99
**POSTOPERATIVE oMEDD (mg)**			
PACU	0 (0:10.5)	6 (0:21)	0.001
POD 0	25.9 (4:60)	46 (15:82)	0.007
POD 1	91.2 (39.6:204.7)	128.6 (79.0:187.3)	0.12
POD 1 (cumulative including POD 0)	125.7 (52.5:268.1)	176.3 (105:269.5)	0.04
POD 2	95.7 (44.7:164.5)	90 (51.1:165.7)	0.71
POD 2 (cumulative including POD 0–1)	231.1 (116.3:420.2)	255 (177:432.8)	0.14
POD 3	57 (30:104)	67.5 (30:116)	0.57
POD 3 (cumulative including POD 0–2)	283.6 (164.1:526.3)	343.5 (223.4:498)	0.13
**POSTOPERATIVE KETAMINE**			
Patients receiving ketamine in the PACU	6 (4.8%)	3 (3.3%)	0.74
Median amount (mg)	10 (8.75:21)	19.8 (10:30)	0.45
Patients receiving ketamine on POD 0	57 (45.6%)	27 (29.7%)	0.02
Median amount (mg)	30 (19.5:48)	64 (35.8:96)	0.006
Patients receiving ketamine on POD 1	59 (47.2%)	36 (39.6%)	0.27
Median amount (mg)	0 (0:96)	0 (0:130)	0.78
Patients receiving ketamine on POD 2	42 (33.6%)	27 (29.7%)	0.56
Median amount (mg)	0 (0:48)	0 (0:54)	0.71
Patients receiving ketamine on POD 3	19 (15.2%)	15 (16.5%)	0.85
Median amount (mg)	0 (0:0)	0 (0:0)	0.67
Patient controlled analgesia use (hours)	47.5 (40:71)	63.5 (42.25:71.5)	0.33

### Postoperative opioid requirements

In the post anaesthesia care unit, the median (IQR) cumulative oMEDD requirement was lower in the ITM group: 0 mg (0:10.5) compared to the usual care group 6 mg (0:21), *p* = 0.001. Postoperative opioid requirements were lower in the ITM group on postoperative Days 0 to 1*.* No statistically significant differences were observed in cumulative oMEDD requirements between the ITM group and the usual group on postoperative days 2 and 3. For patients receiving ITM, there were no significant differences observed between the dose of ITM administered and the incidence of nausea and vomiting requiring anti-emetics (150-200 μg vs. 300 μg, *p* = 0.611; 300 μg vs. 400-500 μg, *p* = 0.272; 150-200 μg vs. 400-500 μg, *p* = 0.098). Further, there were no significant differences observed in oMEDD use on postoperative day 1 in ITM patients who were administered other analgesic adjuncts compared to those who received ITM alone.

The median (IQR) postoperative ketamine requirement was lower in the ITM group on postoperative day 0: 30 mg (19.5:48) vs. 64 mg (35.8:96) in the usual care group, *p* = 0.006. No statistically significant differences in postoperative ketamine requirements were identified at other timepoints. A detailed overview of intraoperative outcomes and postoperative opioid and ketamine requirements is presented in Table 3.

### Postoperative analgesia

In the PACU, the median (IQR) pain score at rest was 1 (0:3) in the ITM group compared to 4 (0:7) in the usual care group, *p* = 0.001. On postoperative day 0, median (IQR) pain scores at rest in the ITM group were 3 (0:5) vs. 5 (3:7) in the usual care group, *p* = 0.003. No statistically significant difference in pain scores at rest on postoperative days 1–3 were observed. Median (IQR) pain scores on movement on postoperative day 0 was 3 (1:5) in the ITM group vs. 4 (3:6) in the usual care group, *p* = 0.007. No statistically significant differences in pain scores on postoperative days 2 and 3 between the groups were identified. For patients receiving ITM, there were no significant differences in pain scores on movement or at rest between patients receiving 150-200 μg, 300 μg or 400-500 μg of ITM.

### Postoperative outcomes and complications

No statistically significant differences were identified between the ITM group and usual care group in time to full ward diet, time to first oral opioid use, complications, length of stay and 30-day readmission. Patients in the ITM group had a longer median (IQR) stay in the ICU: 17 h (12:21.5) vs. 10 h (0:18), *p* = 0.0001. Patients in the usual care group had a greater incidence of severe sedation on postoperative day 0 (42.4% vs 28.8%, *p* = 0.04). A detailed overview of postoperative and complication outcomes for each study group is presented in Table 4. For patients receiving ITM, there were no significant differences observed in the severity or number of complications between patients receiving 150-200 μg, 300 μg or 400-500 μg of ITM. No significant changes were observed in the length of hospital stay over time period 2011 to 2017 (*P* = 0.153 Kruskal-Wallis).

**Table 4:** Postoperative outcomes of patients receiving intrathecal morphine (ITM) or Usual care. Data presented as median (interquartile range) or number of patients (proportion)

	ITM (***n*** = 125)	Usual care (***n*** = 91)	***P***-value
Time in the intensive care unit (hours)	17 (12:21.5)	10 (0:18)	0.0001
Time to full ward diet (hours)	79 (54.5:116.8)	73.75 (50.75:102.8)	0.41
Time to first oral opioid use (days)	2 (2:3)	2 (1:3)	0.98
Patients with at least one complication	88 (70.4%)	67 (73.6%)	0.65
**NUMBER OF COMPLICATIONS**			
0	37 (29.6%)	24 (27.0%)	0.90
1	31 (24.8%)	25 (28.1%)	
2	16 (12.8%)	11 (12.4%)	
3	9 (7.2%)	9 (10.1%)	
≥4	32 (25.6%)	20 (22.5%)	
**WORST CLAVIEN-DINDO GRADE OF COMPLICATIONS**			
I	26 (29.5%)	22 (33.8%)	0.13
II	51 (58.0%)	28 (43.1%)	
III	5 (5.7%)	8 (12.3%)	
IV	4 (4.5%)	7 (10.8%)	
V	2 (2.3%)	0 (0%)	
Respiratory depression requiring naloxone	1 (0.8%)	1 (1.1%)	> 0.99
Nausea or vomiting requiring anti-emetics	101 (80.8%)	72 (79.1%)	0.86
Pruritis requiring treatment with antihistamine, 5-hydroxytryptamine or dopamine receptor antagonist, or opiate-antagonist	17 (13.6%)	9 (9.9%)	0.53
**SEVERE SEDATION**			
POD 0	36/125 (28.8%)	38/90 (42.2%)	0.04
POD 1	19/122 (15.6%)	13/87 (14.9%)	0.99
POD 2	5/107 (4.7%)	9/75 (12.0%)	0.09
POD 3	1/25 (4%)	3/55 (5.5%)	0.99
Length of stay (days)	6 (5:10)	7 (5:10)	0.69
30-day readmission	11 (8.8%)	9 (9.9%)	0.82
Time in the intensive care unit (hours)	17 (12:21.5)	10 (0:18)	0.0001
Time to full ward diet (hours)	79 (54.5:116.8)	73.75 (50.75:102.8)	0.41
Time to first oral opioid use (days)	2 (2:3)	2 (1:3)	0.98
Patients with at least one complication	88 (70.4%)	67 (73.6%)	0.65

### Postoperative cost analysis

Median (IQR) total hospital costs for all patients were US$11,183 (8458:17,331). Costs relating to blood products, medical complications, MET calls, pharmacy, radiology and ward were similar between the ITM and the usual care group. Costs relating to allied health, ICU and pathology were higher in the ITM group. The median (IQR) costs for allied health in US$ were $454 (362.7:933.8) in the ITM group vs. $366.6 (116.5:907.8) in the usual care group, *p* = 0.002; Median (IQR) total hospital costs (US$) were higher in the ITM group: $11,640 (9106:17,247) vs. $10,338 (7419:18,664), *p* = 0.05. A detailed overview of postoperative costs for each study group is presented in Table 5.

**Table 5:** Postoperative costs in US dollars of patients receiving intrathecal morphine (ITM) or Usual care. Data presented as median (interquartile range)

Cost centre	ITM (***n*** = 125)	Usual care (***n*** = 91)	***P***-value
**Allied health**	$454 (362.7:933.8)	$366.6 (116.5:907.8)	0.002
**Blood products**	$171.4 (120.3:1012)	$1258 (92.46:3107)	0.21
**Intensive care unit**	$2385 (1692:3355)	$1600 (0:3375)	0.001
**Medical**	$1470 (1047:1945)	$1638 (1205:2318)	0.06
**Medical emergency team**	$216.8 (201.8:403.6)	$433.5 (263.5:605.4)	0.11
**Pathology**	$1227 (795.7:1804)	$898.4 (521:1440)	0.003
**Pharmacy**	$359.6 (280.1:518.5)	$344.1 (232.1:443)	0.08
**Radiology**	$312.6 (142:1016)	$231.6 (101.3:953.3)	0.65
**Ward**	$4516 (3605:6935)	$4824 (3781:6653)	0.35
**Grand total**	$11,640 (9106:17,247)	$10,338 (7419:18,664)	0.05

## Discussion

### Key findings

We performed a single-centre observational study evaluating the opioid sparing and analgesic effects of ITM on adult patients undergoing open liver. As hypothesized, for patients undergoing open liver resection using a standardized enhanced recovery after surgery protocol with multimodal analgesia, the addition of ITM resulted in less postoperative opioid use and enhanced postoperative analgesia. Further, we found no statistically significant differences in opioid-related complications, time to mobilisation, and length of hospital stay. Hospital costs were significantly higher in patients receiving ITM, reflective of a longer mandatory stay in ICU. Our findings support the use of single shot intrathecal morphine as an efficacious analgesic technique in patients undergoing open hepatic resection.

Our findings of reduced oMEDD use at 24 h after surgery in the ITM group are congruent with other studies reporting that ITM reduces postoperative opioid requirements compared to conventional analgesia strategies for up to 24 h after surgery, but not beyond [8, 16, 17]. Thus ITM use provided statistically significant opioid sparing and analgesic benefits without any increase in opioid related side effects such as sedation and delayed respiratory depression, findings previously reported with high-doses of systematically administered opioids [18]. Interestingly, our findings revealed that ITM use significantly reduced postoperative opioid consumption at 24 h postoperatively in the 25th and 50th quartiles, implying the greatest benefit in this cohort of patients. Identifying these patients preoperatively remains challenging in the present time, however with modern computational functional genomics being rapidly developed, it may be possible to exploit genetic information from increasingly available data sets for patients with complex diseases, such as pain and liver cancer patients, that in turn may offer a new insight into which patients may benefit most from ITM therapy as part of complementary patient and surgery centric approaches.

Interestingly, postoperative pain scores shared the same temporal pattern as oMEDD use at 24 h after surgery, and pain was significantly reduced by ITM up to 24 h after surgery, but not beyond. The medical literature evaluating ITM analgesia in open liver resection, as well as in other procedures including thoracotomy and caesarian section, mirror our findings of ITM enhancing analgesia up to 24 h post operation [8, 16, 17, 19]. It is well-known that the analgesic effect of ITM can last up to 24 h post operation with a concomitant decrease in supplementary opioid requirement during this period [20, 21].

With respect to other postoperative outcomes, our findings reflect the array of studies which have universally reported that ITM causes no statistically significant difference in length of stay in open liver resection compared to other analgesic modalities [8, 16, 17, 19]. Similarly our findings were congruent with others reporting no differences in complication rate and complication severity between ITM and other analgesic modalities [8, 16, 17, 19]. No neurological sequelae of intrathecal administration were observed in our study. Concerns that intrathecal opioid use is associated with a higher incidence of opioid-related side effects such as sedation and respiratory depression were unfounded by our results. Among these side effects, respiratory depression is the most feared. Our findings are further supported by a meta-analysis of 28 studies for a range of surgical procedures, which found that there was no increased risk of respiratory depression with low-dose ITM (≤400 μg) compared to systemic opioids [22]. Nonetheless, ITM does need to be used with caution in elderly patients (> 80 years of age), patients with chronic respiratory and renal impairment, and patients with obstructive sleep apnoea [23]. As an additional safety precaution against delayed-onset respiratory depression, our hospital protocol mandates a 24-h stay in the ICU following surgery involving ITM analgesia to monitor for respiratory depression via the ETCO_2_, PaO_2_, PaCO_2_, respiratory rate and oxygen saturation measurements [17, 23]. Reflective of this mandatory ICU stay, we showed that total postoperative hospital costs were increased in the ITM group.

### Strengths and limitations

There are several strengths and limitations of this study. To date this is the largest review of ITM use in the context of open hepatic resection. A previous study assessing ITM in open liver resection was conducted by Sakowska et al. and involved 161 participants - less than half the size of our study [6]. Furthermore, our study collected comprehensive data detailing intraoperative and postoperative analgesia requirements, postoperative pain scores, and postoperative complications. Previous studies have been limited by failing to comprehensively report major outcomes, rather focusing on minor postoperative complications such as nausea, vomiting and sedation [8, 16, 23, 24]. In comparison, we have also detailed the incidence of all major systemic complications. Finally, to our knowledge, no other study has examined the costs of ITM in open liver resection compared to conventional analgesic modalities.

This is a single-centre study performed in a high-volume hepatobiliary unit within a tertiary healthcare centre, partly limiting the external validity of our findings. However, our hospital has all the typical characteristics of many tertiary institutions’ hepatobiliary units, and the surgical and anaesthesia perioperative protocols adopted by our centre are aligned with those in many other tertiary centres. All patients were adults who underwent open hepatic resection, which also limits the generalisability of our findings to paediatric liver resections, and to adult patients undergoing other types of surgeries. Given the retrospective nature of the study, we cannot establish a causal relationship between ITM and the change in the perioperative variables we assessed.

Another significant limitation of our findings is that given the retrospective nature of our study, the collection of data may have been subject to human error in the interpretation and recording of data. However, we consider this an unlikely source of error given the comprehensive cross-checks required for data entry at our institution and the use of electronic medical records. Additionally, the data collection was conducted by a clinician not involved in postoperative patient care, thereby minimising the likelihood of detection bias. Being a retrospective observational study with no possibility of randomisation or subject blinding, our study lacks in validity compared to a randomized controlled trial. Nonetheless the large sample size, in addition to the congruence of our results with the existing medical literature, lends significant strength to our study. Our findings are hypothesis generating and may provide valuable data for power calculations for future studies on evaluating the effects of ITM on perioperative oMEDD use, analgesia, and various adverse outcomes. Finally, validation of our findings with a large multi-centre RCT, similar in design to the Multicenter Australian Study of Epidural Anesthesia Trial which involved 25 hospitals in Australia and South-East Asia, can be justified [4].

## Conclusion

In patients undergoing open liver resection, ITM in addition to conventional multimodal analgesic strategies reduced postoperative opioid requirements and improved analgesia for 24 h after surgery, without any statistically significant differences in opioid-related complications, time to mobilisation, and length of hospital stay. Hospital costs were significantly higher in patients receiving ITM, reflective of a longer mandatory stay in ICU. Our findings support the use of single shot intrathecal morphine as an efficacious analgesic technique in patients undergoing open hepatic resection. A RCT evaluating the effects of ITM in addition to conventional multimodal analgesic is justified.

## Data Availability

The data that support the findings of this study are available from the corresponding author upon reasonable request.

## Abbreviations

ANZCTR: Australian New Zealand Clinical Trials Registry
ASA: American Society of Anesthesiologists
BMI: Body mass index
CCI: Charlson Comorbidity Index
ERAS: Enhanced recovery after surgery
ETCO_2_: End tidal carbon dioxide
HREC: Health Human Research Ethics Committee
ICD: International Statistical Classification of Diseases
ICU: Intensive care unit
IQR: Interquartile range
ITM: Intrathecal morphine
NRS: Numerical Rating Scale
oMEDD: Oral morphine equivalent daily dose
PACU: Post-anaesthesia care unit
PaO_2_: Partial pressure of oxygen
PaCO_2_: Partial pressure of carbon dioxide
POD: Postoperative day
RCT: Randomized controlled trial
